# Low dose chloroquine decreases insulin resistance in human metabolic syndrome but does not reduce carotid intima-media thickness

**DOI:** 10.1186/s13098-019-0456-4

**Published:** 2019-07-29

**Authors:** Janet B. McGill, Mariko Johnson, Stacy Hurst, William T. Cade, Kevin E. Yarasheski, Richard E. Ostlund, Kenneth B. Schechtman, Babak Razani, Michael B. Kastan, Donald A. McClain, Lisa de las Fuentes, Victor G. Davila-Roman, Daniel S. Ory, Samuel A. Wickline, Clay F. Semenkovich

**Affiliations:** 1 Division of Endocrinology, Metabolism & Lipid Research, Department of Medicine, Washington University School of Medicine, 660 South Euclid Avenue, Box 8127, St. Louis, MO 63110 USA; 2 Program in Physical Therapy, Washington University, St. Louis, MO USA; 3 Division of Biostatistics, Washington University, St. Louis, MO USA; 4 Cardiovascular Division, Washington University, St. Louis, MO USA; 5 Department of Pharmacology & Cancer Biology, Duke University, Durham, NC USA; 6 Department of Internal Medicine, Wake Forest School of Medicine, Winston-Salem, NC USA; 7 Department of Cell Biology & Physiology, Washington University, St. Louis, MO USA

**Keywords:** Chloroquine, Metabolic syndrome, Carotid intima-media thickness, Insulin sensitivity, Glucose disposal, Atheroma, Blood pressure, Lipids, JNK

## Abstract

**Background:**

Metabolic syndrome, an obesity-related condition associated with insulin resistance and low-grade inflammation, leads to diabetes, cardiovascular diseases, cancer, osteoarthritis, and other disorders. Optimal therapy is unknown. The antimalarial drug chloroquine activates the kinase ataxia telangiectasia mutated (ATM), improves metabolic syndrome and reduces atherosclerosis in mice. To translate this observation to humans, we conducted two clinical trials of chloroquine in people with the metabolic syndrome.

**Methods:**

Eligibility included adults with at least 3 criteria of metabolic syndrome but who did not have diabetes. Subjects were studied in the setting of a single academic health center. The specific hypothesis: chloroquine improves insulin sensitivity and decreases atherosclerosis. In Trial 1, the intervention was chloroquine dose escalations in 3-week intervals followed by hyperinsulinemic euglycemic clamps. Trial 2 was a parallel design randomized clinical trial, and the intervention was chloroquine, 80 mg/day, or placebo for 1 year. The primary outcomes were clamp determined-insulin sensitivity for Trial 1, and carotid intima-media thickness (CIMT) for Trial 2. For Trial 2, subjects were allocated based on a randomization sequence using a protocol in blocks of 8. Participants, care givers, and those assessing outcomes were blinded to group assignment.

**Results:**

For Trial 1, 25 patients were studied. Chloroquine increased hepatic insulin sensitivity without affecting glucose disposal, and improved serum lipids. For Trial 2, 116 patients were randomized, 59 to chloroquine (56 analyzed) and 57 to placebo (51 analyzed). Chloroquine had no effect on CIMT or carotid contrast enhancement by MRI, a pre-specified secondary outcome. The pre-specified secondary outcomes of blood pressure, lipids, and activation of JNK (a stress kinase implicated in diabetes and atherosclerosis) were decreased by chloroquine. Adverse events were similar between groups.

**Conclusions:**

These findings suggest that low dose chloroquine, which improves the metabolic syndrome through ATM-dependent mechanisms in mice, modestly improves components of the metabolic syndrome in humans but is unlikely to be clinically useful in this setting.

*Trial registration* ClinicalTrials.gov (NCT00455325, NCT00455403), both posted 03 April 2007

## Introduction

A combination of increased triglycerides, low HDL cholesterol, hypertension, above normal fasting glucose, and increased waist circumference constitutes the metabolic syndrome [1], which predisposes to diabetes, cardiovascular disease, and all-cause mortality [2]. Current therapy is directed at individual components of the syndrome, which are related to insulin resistance. Obesity-related insulin resistance is associated with low-grade systemic inflammation characterized by increased activation of a stress kinase known to induce insulin resistance, c-Jun N-terminal kinase (JNK) [3]. Pronounced weight loss is associated with decreased cardiovascular events [4], but there are other causes of insulin resistance besides obesity that could provide insight into relationships between insulin resistance and cardiovascular disease. DNA damage disorders including Hutchinson–Gilford progeria, Werner syndrome, Cockayne syndrome, and ataxia telangiectasia are associated with insulin resistance and vascular disease [5]. Mice deficient in ataxia telangiectasia mutated (ATM), the kinase mutated in ataxia telangiectasia, have increased activation of JNK in macrophages, insulin resistance, hypertension, and increased atherosclerosis [6–8]. ATM activation by the anti-malarial drug chloroquine decreases macrophage JNK activation, blood pressure, insulin resistance, and atherosclerosis in mice [6, 9].

Since the ATM axis is associated with altered hepatic insulin sensitivity and atherosclerosis in mice, we translated these rodent data to humans with metabolic syndrome in two exploratory clinical trials: (1) A dose escalation study with a primary endpoint of insulin sensitivity and pre-specified secondary endpoints including serum lipids and blood pressure; and (2) Placebo or chloroquine treatment for 1 year with a primary endpoint of carotid intima-media thickness, a noninvasive predictor of cardiovascular events [10]. The 1 year study utilized the lowest dose associated with improved insulin sensitivity in the dose escalation study and included pre-specified secondary endpoints such as carotid MRI, activation of JNK in isolated monocytes, serum lipids, and blood pressure.

## Subjects and methods

### Subjects and trial design

These single center studies were approved by the Washington University Human Research Protection Office. All subjects gave informed consent and were compensated for their participation. A data safety and monitoring board convened by the National Heart, Lung and Blood Institute (NHLBI) supervised the trials.

For both the dose escalation and double blind trials, inclusion criteria included men and women of any ethnic group between the ages of 18–60 (Trial 1), and 18–70 (Trial 2) (the difference in age between protocols was intended to make the randomized trial more pertinent to a broader age group) who had at least 3 of the components of the metabolic syndrome: elevated fasting triglycerides (≥ 1.69 mmol/L); low HDL cholesterol (< 1.29 mmol/L in women, < 1.03 mmol/L in men); hypertension (≥ 130/85 mm Hg ≤ 160/100 mmHg) untreated, or hypertension controlled (≤ 150/90 mm Hg) on a stable medication regimen; increased waist circumference (> 89 cm in women, > 102 cm in men); elevated fasting glucose (≥ 5.6 mmol/L and < 7.0 mmol/L). For a diagnosis of hypertension, measurements were repeated on two separate days. Fasting glucose was also repeated on two separate days. Fasting lipids were performed once before enrollment but repeated throughout the interventions.

Exclusion criteria were extensive and included: known prior treatment with chloroquine or hydroxychloroquine, BMI > 45 kg/m^2^, coronary artery disease or other vascular disease, history of stroke, eGFR (estimated glomerular filtration rate) < 60 ml/min/1.73 m^2^, diabetes, seizure disorder, history of psoriasis, hematological disorders (including anemia), current malignancy, asthma or COPD (chronic obstructive pulmonary disease), liver disease (including transaminases > 2× upper limit of normal), active infection (including HIV-human immunodeficiency virus), any serious illness requiring ongoing medical care, major psychiatric illness, lipid lowering medications (other than statins and < 1 g daily fish oils), severe hypertension at baseline or taking more than three antihypertensives, use of cimetidine or vitamin E, pregnancy or lactation or intention to become pregnant, inadequate use of contraception, glucose-6-phosphate dehydrogenase deficiency, auditory disease or hearing loss, retinal disease (especially presence of drusen or pigmentary changes at the macula), and any ocular disease interfering with the ability to rigorously assess the retina.

**Fig. 1:**
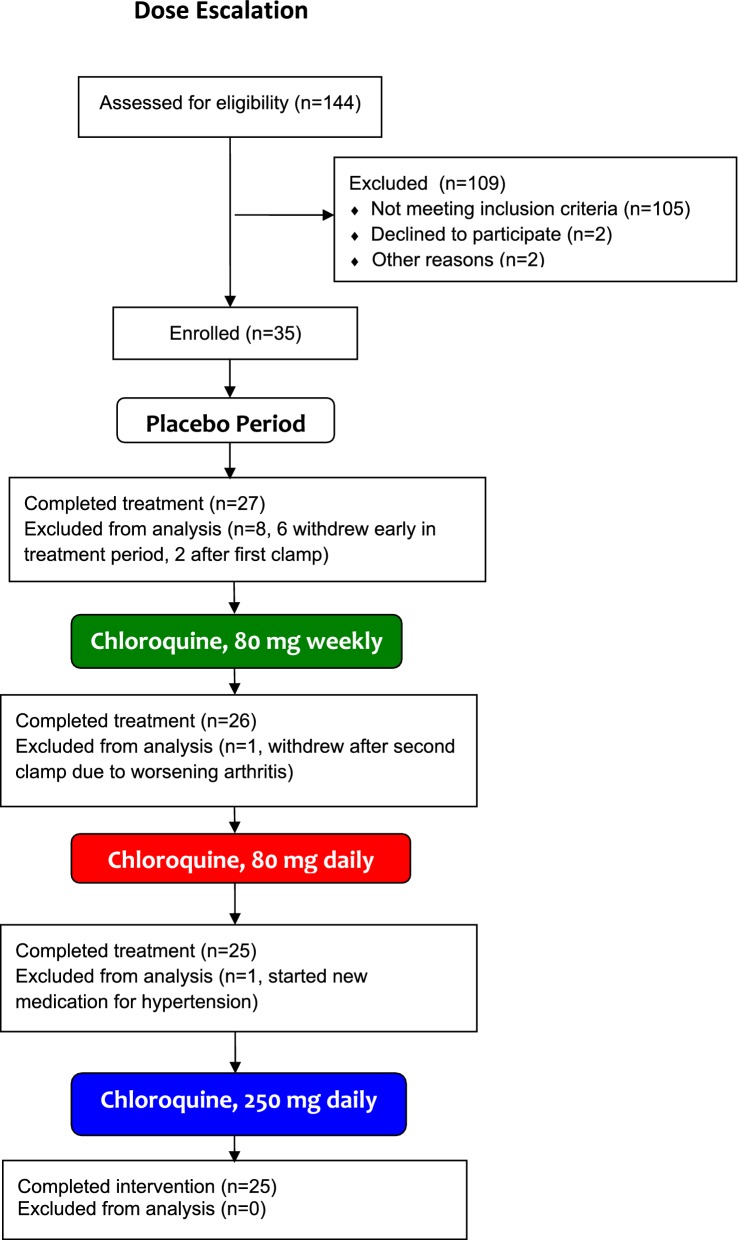
CONSORT statement flow diagram for subjects in dose escalation study

**Fig. 2:**
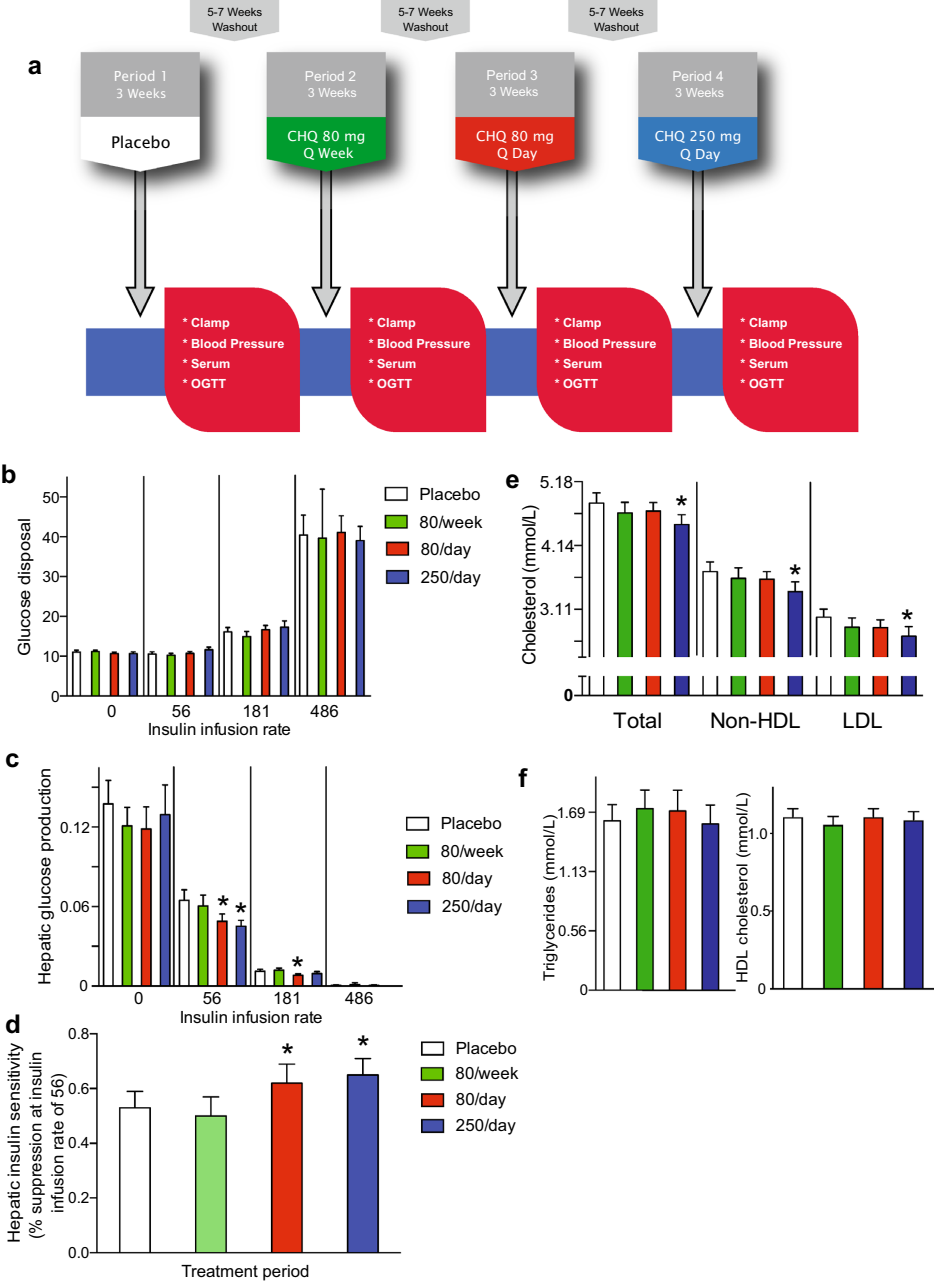
Chloroquine dose escalation trial design and results for hyperinsulinemic-euglycemic clamping and lipids. **a** Diagram of the dose escalation protocol. A 5–7 week washout period was required between limbs to allow for hematologic recovery following the blood drawing associated with clamps. **b** Glucose disposal rates expressed as μmol glucose/kg body weight/min. Insulin infusion rates were 0, 56, 181, or 486 pmol/m^2^/min. **c** Hepatic glucose production expressed as (μmol glucose/kg body weight/min) per pmol/L insulin. **d** Hepatic insulin sensitivity expressed as percent suppression of glucose production at 56 pmol/m^2^/min. **e** Total, non-HDL, and LDL cholesterol at the end of each limb. **f** Triglycerides and HDL cholesterol at the end of each limb. Data represent mean ± SE. *P < 0.05 by Tukey’s test for multiple comparisons after repeated measures ANOVA

A CONSORT statement flow diagram for the first trial is shown in Fig. 1. The study design for the first trial is shown in Fig. 2a. One hundred forty-four subjects were screened for participation in Trial 1 and 35 individuals with the metabolic syndrome qualified. Twenty-five subjects completed the protocol. Ten discontinued the study for reasons that included: starting a new medication during the protocol [3], new diagnosis of diabetes during the protocol [1], difficult IV access [1], worsening joint symptoms [1], and miscellaneous issues unrelated to the protocol (such as relocation, 4). For this single blind, placebo-controlled study, each subject took a capsule that was identical in appearance for 21 days. For the first limb, capsule contents were inert. For the second limb, three of the 21 capsules (administered at weekly intervals) contained 80 mg of chloroquine and the remaining capsules were inert. For the third and fourth limbs, capsules contained 80 or 250 mg of chloroquine, respectively. Each limb was separated by a washout period to allow recovery from the blood drawing of the clamp procedure. It was not possible to randomize limb order due to the extremely long half-life of chloroquine; ingestion during an early limb would be associated with tissue levels of the drug and its metabolites during a later placebo limb.

**Fig. 3:**
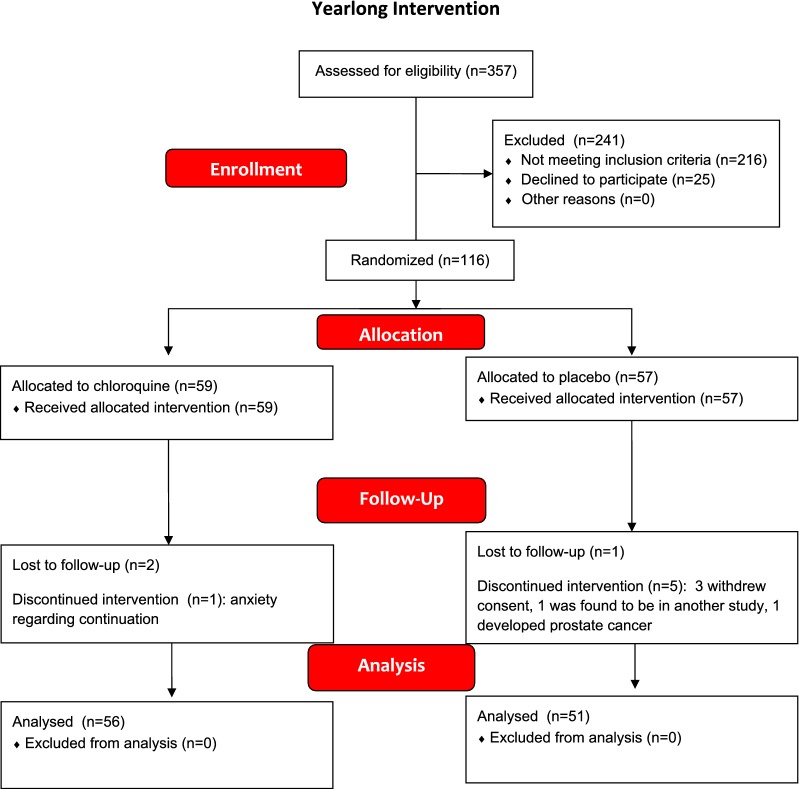
CONSORT statement flow diagram for subjects in the yearlong randomized clinical trial

**Fig. 4:**
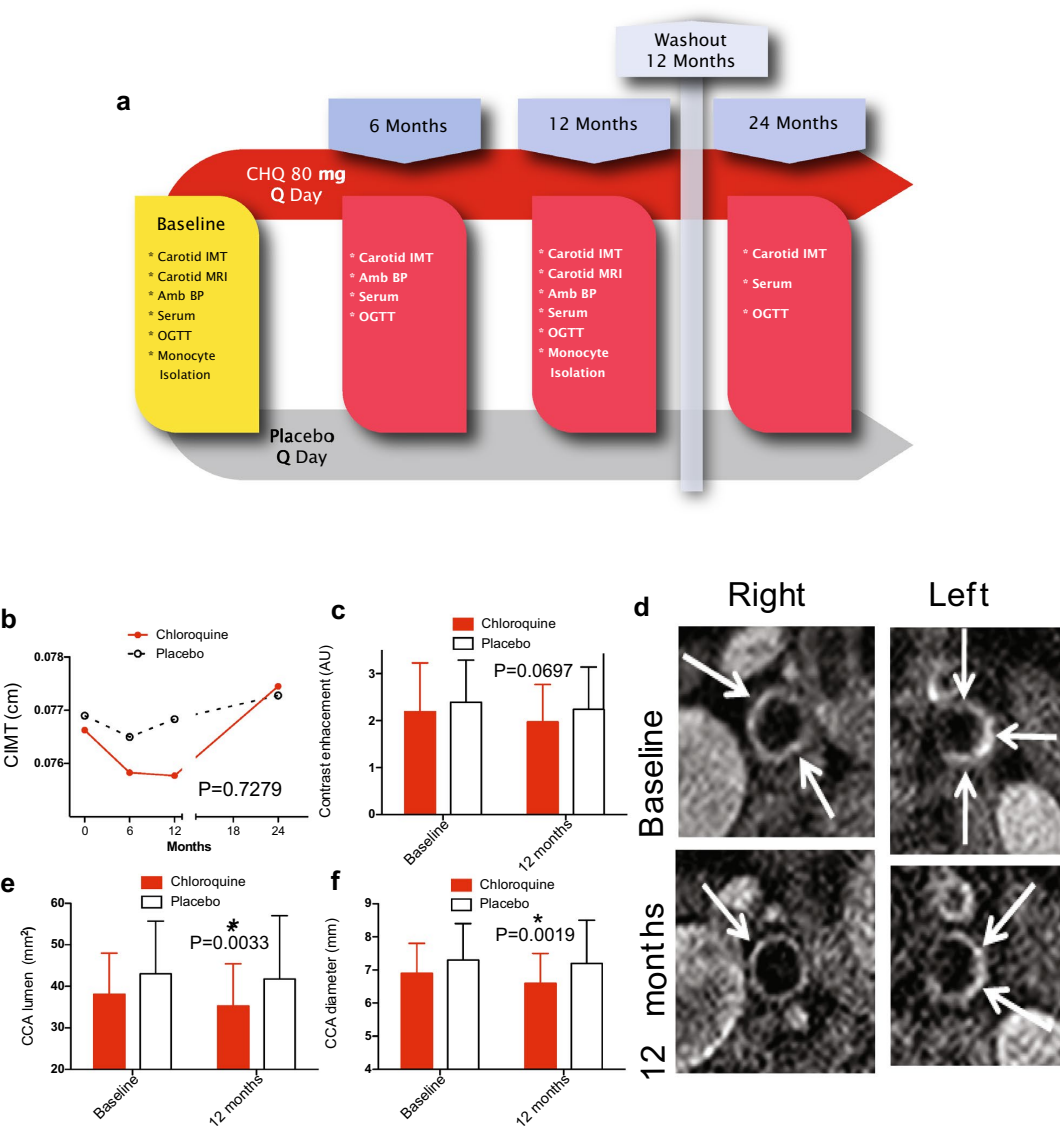
Yearlong chloroquine trial design and results for carotid imaging. **a** Diagram of the vascular endpoint trial. After 12 months of placebo or chloroquine, both were stopped, then subjects returned at 24 months for limited additional studies. **b** CIMT results. Standard deviations (not included to simplify data presentation) and n values for these data follow. Chloroquine: 0, 0.76625 ± 0.17270 (n = 56); 6 months, 0.75827 ± 0.17634 (n = 56); 12 months, 0.75769 ± 0.16061 (n = 54); 24 months, 0.77450 ± 0.16540 (n = 53). Placebo: 0, 0.76900 ± 0.11653 (n = 53); 6 months, 0.76500 ± 0.13087 (n = 51); 12 months, 0.76833 ± 0.11487 (n = 50); 24 months, 0.77280 ± 0.13290 (n = 49). **c** MRI-determined contrast enhancement. P = 0.0697. **d** Representative images at baseline and at 12 months for the same vessels. **e** MRI-determined common carotid lumen area *P = 0.0033. **f** MRI-determined common carotid artery diameter. *P = 0.0019. Data represent mean ± SD in **c**, **e**, **f**. Analysis by mixed model testing in **b**, paired t tests in **c**, **e**, **f**

A CONSORT statement flow diagram for the second trial is shown in Fig. 3. The study design for the second trial is shown in Fig. 4a. The blind was held by a research pharmacist, who provided the randomization sequence to the blinded research nurses or physicians who enrolled participants and assigned interventions that included study medications that were identical in appearance. Three hundred fifty-seven subjects were screened for participation in Trial 2 and 155 non-diabetic individuals with metabolic syndrome qualified at the initial screening visit. Of these, 21 withdrew consent before the second screening visit, 4 withdrew consent after the second screening visit and 14 did not qualify for randomization due to abnormal eye exams [4], abnormal carotid imaging [2], anemia [3], G6PD deficiency [2], abnormal liver function tests [2], and psoriasis [1]. The remaining 116 subjects were randomized to placebo or chloroquine. Of those randomized, 19 assigned to placebo and 20 assigned to chloroquine were being treated for hypertension, and were taking 1.3 ± 1.1 and 1.1 ± 1.0 antihypertensive medications respectively. 15 subjects in each group were taking a statin.

### Hyperinsulinemic–euglycemic clamp procedure

At the end of each limb depicted in Fig. 2a, subjects presented after an overnight fast. The antecubital vein was catheterized for infusion of insulin, glucose and tracer solutions, a hand vein was catheterized for blood sampling, and the hand was maintained in a hotbox at ~ 55 °C to arterialize venous sampling. Baseline samples were obtained prior to initiating infusions.

Insulin sensitivity was assayed using a two-step euglycemic clamp technique as described previously [11]. The stable isotope [6,6-^2^H_2_] glucose (22 μmol/kg prime and 0.25 μmol/kg/min constant infusion) was infused during a 90 min basal period (to allow isotope equilibration) and then continued throughout each study. Plasma glucose was measured at 5–10 min intervals following the basal period. Insulin was initially infused in two stages consisting of rates of 181 and 486 pmol/m^2^/min, but after analyzing results from the first 7 patients, the protocol was modified to include two stages consisting of insulin infusion rates of 56 and 181 pmol/m^2^/min in order to avoid nearly complete suppression of hepatic glucose production at higher insulin infusion rates. Twenty % dextrose (containing 1.5% [6,6-^2^H_2_] glucose) was infused to maintain blood glucose at 5.00 mmol/L for ~ 2 h at each stage. Plasma was assayed for 6,6-^2^H_2_-glucose enrichment by mass spectrometry. Glucose kinetics were determined using samples obtained at 10 min intervals during the last 30 min of the basal period and during each stage of the clamp.

### Glucose tolerance testing and assays of serum or plasma

Oral glucose tolerance testing was performed by administering 75 g of glucose followed by sampling at 0, 30, 60, 90 and 120 min. Samples were assayed for glucose, insulin, C peptide, and glucagon. AUC calculations were performed using the trapezoid rule for approximating integrals. Lipids, lipoproteins, serum chemistries, and blood counts were assayed by the Washington University Core Laboratory for Clinical Studies, which is CLIA-certified. Since this project addressed the effects of chloroquine on insulin resistance, the cause of which remains obscure, the laboratory also performed exploratory measurements of putative metabolic mediators that have been implicated in insulin resistance and/or its vascular complications [12–16]. These included adiponectin, leptin, iron, VEGF (vascular endothelial growth factor), and IL-17A. Samples for adiponectin and iron studies were assayed at the University of Utah [17].

### Blood pressure determinations

Two techniques were employed: auscultation of seated subjects at rest was performed by a trained observer who recorded the first and fifth phases of the Korotkoff sounds; and, a portable oscillometric device (SpaceLabs Medical) recorded results every 20 min during the day and every h during the night then data were analyzed as mean values over 24 h, between 0800 and 2200 (daytime), and between 2200 and 0800 (nighttime).

### CIMT assessments

Carotid artery intima-media thickness (CIMT) was measured from B-mode images by a single sonographer using standard approaches [18, 19]. The average of 3 measurements of the posterior wall of each common carotid ~ 1 cm proximal to the carotid bulb was determined; plaques (wall thickening > 50% of the surrounding wall) were excluded. To establish reproducibility, 10% of the images were randomly selected and re-measured in a blinded fashion by two independent observers. Intra-observer and inter-observer correlation coefficients were 0.900 and 0.992.

### MRI assessments

A contouring tool on the Philips ViewForum workstation was used to outline the inner and outer common carotid artery wall regions (right and left) in the first frame immediately below the bifurcation in the T2-weighted image, then areas and diameters of the vessel wall and lumen were calculated. The maximum thickness of the common carotid in the same frame was measured with the line tool. Similar measurements were made for the internal carotid artery in a slice just beyond the bifurcation. For common carotid artery wall contrast enhancement, which represents gadolinium leakage and late hyper enhancement, pre- and post-injection T1 weighted images were compared by dividing the artery into quadrants. The slice nearest the bifurcation manifesting the greatest signal in terms of numbers of quadrants enhanced was chosen by visual comparison with the same pre-injection slice. If the pre-injection signal in the carotid wall appeared bright in any quadrant due to signal spatial heterogeneity, and with reference to the other arterial wall segments in adjacent or contralateral arteries, then the post- injection signal was deemed nonenhancing. Concurrence of two readers was achieved in blinded interpretations.

### Monocyte protein preparation, JNK assays, and oxysterol determinations

Human monocytes were isolated using a negative selection technique employing a RosetteSep Human Monocyte Enrichment Cocktail (StemCell Technologies #15028), exactly as described by the manufacturer. Cells were lysed using a buffer containing 1% Tween 20 and 0.2% NP40 with protease and phosphatase inhibitors, and lysates were aliquoted and stored at − 80 °C.

Total and phospho (Thr183/Tyr185) JNK were assayed using chemiluminescent sandwich ELISA kits (Cell Signaling Technologies #7869S and #7849S). Extracts from subjects before and 1 year following treatment were analyzed in the same assay by an observer masked to randomization status. Signals detected from 1 μg lysate protein (assayed with BCA reagents from Pierce) were determined using a Synergy 4 plate reader and the ratio of phospho-JNK to total JNK was calculated. For assays of cholestane-3β,5α,6β-triol, plasma samples stored at − 80 °C were thawed and combined with BHT (50 μg/mL). Oxysterols were purified, derivatized, then quantified by GC–MS (*m/z* 456) in assays performed within the linear response range of appropriate standard curves [20].

### Statistical analyses

Results are expressed as mean ± SD except in Fig. 2 where results are presented as mean ± SE to simplify data presentation. Statistical analyses were performed using GraphPad Prism software. For the dose escalation study, most data were analyzed using repeated measures ANOVA with post hoc testing using Tukey’s multiple comparison test for inter-group differences. For the yearlong treatment study, most data were analyzed using a mixed model approach for repeated measures to allow for dealing with missing values. P values for mixed model analyses represent an assessment of whether the dependent variable was affected by the randomization group (placebo vs. chloroquine) vs. time interaction. Some analyses in both trials involving only two groups were performed using a two-tailed Student’s t test. Spearman correlation coefficients were calculated for the relationships between baseline CIMT in the 116 randomized subjects and variables associated with atherosclerosis. Two-tailed paired t tests were used to analyze the baseline vs. 12-month results for MRI imaging and JNK activation. Binary baseline characteristics and adverse events were analyzed using contingency tables and Chi Square Test or Fisher’s Exact Test.

## Results

### Metabolic effects of increasing doses of chloroquine

Characteristics for those completing the dose escalation study are shown in Table 1. The primary endpoint of the first trial was clamp-determined insulin sensitivity. Two-stage hyperinsulinemic euglycemic clamps were initially performed using insulin at 181 and 486 pmol/m^2^/min. However, data analysis after the first seven patients demonstrated complete suppression of glucose production at the higher dose and nearly complete suppression at the lower dose, decreasing the ability to detect differences in endogenous glucose production in this group of patients with metabolic syndrome but not diabetes. Subsequent two-stage clamps were performed using insulin infusions at 56 and 181 pmol/m^2^/min. The results from these first seven patients are included in Fig. 2b, c; the 486 pmol/m^2^/min condition represents data points from these first patients for each limb while the 181 pmol/m^2^/min condition includes data points from these patients in addition to those from those undergoing clamps at 56 and 181 pmol/m^2^/min. There were no differences in glucose disposal rates between any of the chloroquine doses as shown in Fig. 2b.

**Table 1:** Baseline characteristics of subjects in the dose escalation study

Variable	Results
Number	25
Gender (F/M)	18/7
Race	
Black	2
Non-hispanic white	23
Weight (kg)	101 ± 17
BMI (kg/m^2^)	35.0 ± 4.4
Waist circumference (cm)	109 ± 13
Fasting glucose (mmol/L)	5.27 ± 0.61
Systolic blood pressure (mmHg)	135 ± 16
Diastolic blood pressure (mmHg)	80 ± 10
Total cholesterol (mmol/L)	5.25 ± 0.67
Non-HDL cholesterol (mmol/L)	4.11 ± 0.62
LDL cholesterol (mmol/L)	3.23 ± 0.57
Triglycerides (mmol/L)	1.85 ± 0.61
HDL cholesterol (mmol/L)	1.19 ± 0.26

Data represent mean ± SD

There was a dose-dependent effect of chloroquine on hepatic glucose production (clamp-determined rate of appearance) with significant suppression at doses of 80 mg/day and 250 mg/day as determined by repeated measures ANOVA (Fig. 2c). Figure 2d shows that chloroquine increased hepatic insulin sensitivity based on data from the 56 pmol/m^2^/min stage.

There was also an apparent dose-dependent effect of chloroquine on fasting levels of total cholesterol, non-HDL cholesterol, and LDL cholesterol with a significant decrease at 250/day as determined by repeated measures ANOVA (Fig. 2e). Serum triglycerides and HDL cholesterol were unchanged (Fig. 2f).

Chloroquine in this dose escalation study had no effect on blood pressure determined using an ambulatory monitor as shown in Table 2. SBP, DBP, MAP, and magnitude of BP decrease overnight (“dipping” status) were unaffected in all limbs. Consistent with an effect of chloroquine on hepatic insulin sensitivity, fasting glucose at the time of the clamp was significantly decreased (Table 2). Consistent with the lack an effect of chloroquine on glucose disposal, OGTT AUC results were unaffected in all limbs (Table 2). There was also no effect on body weight, glucagon, leptin, NEFA, C-peptide or insulin (Table 2).

**Table 2:** Dose effect of chloroquine on blood pressure and other variables

Variable	Placebo	80 mg/week	80 mg/day	250 mg/day	P value
SBP (mmHg)	121 ± 12	121 ± 10	123 ± 12	123 ± 12	0.5607
DBP (mmHg)	70 ± 7	71 ± 7	73 ± 8	73 ± 9	0.1107
MAP (mmHg)	87 ± 8	88 ± 7	89 ± 9	90 ± 9	0.1944
Overnight MAP dip	8.8 ± 4.2	9.5 ± 5.3	9.5 ± 8.8	9.6 ± 5.0	0.9878
Fasting glucose clamp	5.77 ± 0.53	5.83 ± 0.52	5.83 ± 0.53	5.44 ± 0.65	0.0010*
Fasting glucose OGTT	5.49 ± 0.57	5.77 ± 0.56	5.72 ± 0.62	5.66 ± 0.62	0.0805
OGTT AUC (mmol/L/h)	16.93 ± 3.05	17.65 ± 3.00	17.87 ± 3.22	17.26 ± 3.61	0.3585
Weight (kg)	104 ± 18	103 ± 18	104 ± 19	103 ± 19	0.6041
BMI	35.9 ± 4.3	35.9 ± 4.2	35.9 ± 4.5	35.5 ± 4.6	0.5964
A1c (%)	5.8 ± 0.4	ND	ND	5.8 ± 0.4	0.9493
Insulin (pmol/L)	153 ± 94	138 ± 62	166 ± 107	148 ± 80	0.4260
C peptide (nmol/L)	1.16 ± 0.44	1.20 ± 0.38	1.26 ± 0.44	1.25 ± 0.36	0.5473
Glucagon (pg/mL)	110 ± 41	101 ± 26	111 ± 54	102 ± 50	0.3646
Leptin (μg/L)	27.5 ± 11.6	27.2 ± 11.1	31.0 ± 16.4	27.2 ± 11.9	0.2134
NEFA (mmol/L)	0.56 ± 0.15	0.55 ± 0.10	0.58 ± 0.19	0.55 ± 0.16	0.8776
TNFα (pg/mL)	12.9 ± 1.8	13.0 ± 1.7	12.2 ± 1.9	12.4 ± 2.0	0.0890
Fibrinogen (μmol/L)	8.44 ± 1.32	8.38 ± 1.47	8.14 ± 1.09	7.94 ± 1.44	0.3563
IL-6 (pg/mL)	1.0 ± 1.6	1.4 ± 1.5	1.2 ± 1.6	0.96 ± 1.3	0.4282
Lp(a) (mg/dL)	22.9 ± 17.5	20.9 ± 16.0	22.5 ± 17.2	22.8 ± 18.1	0.1454
CRP (mg/L)	8.3 ± 10.8	7.9 ± 10.6	6.4 ± 7.2	7.5 ± 8.0	0.7455
Adiponectin (μg/mL)	21.6 ± 8.1	ND	ND	20.6 ± 5.6	0.3503
Iron (μmol/L)	12.3 ± 4.1	ND	ND	11.4 ± 5.5	0.5311
Iron Bind. Cap. (μmol/L)	64.9 ± 8.7	ND	ND	65.8 ± 10.0	0.6385
Transferrin (mg/dL)	300.3 ± 47.5	ND	ND	303.8 ± 48.5	0.6899
Transferrin Sat. (%)	19.1 ± 6.4	ND	ND	17.2 ± 6.9	0.3413
Ferritin (pmol/L)	170 ± 191	ND	ND	105 ± 88	0.0155*

Data represent mean ± SD

*MAP* mean arterial pressure, *NEFA* non-esterified fatty acids, *ND* not determined

P values determined by repeated measures ANOVA except for A1c, adiponectin, and iron studies, where P value determined by paired t test

There was a trend toward lower levels of TNFα (Table 2), but other inflammatory markers including CRP and adiponectin were unaffected (Table 2), consistent with other large studies of the relationship between this adipokine and insulin sensitivity [21].

Iron is linked to the development of diabetes [22] and ferritin is the major storage form of iron. Chloroquine treatment was associated with a significant decrease in ferritin levels in humans (Table 2), confirming an effect seen in rodents [23].

### Vascular effects of chloroquine treatment

Baseline characteristics for the 107 subjects who completed the second trial, a double-blind placebo controlled study are shown in Table 3. The primary endpoint of the second trial was CIMT. To validate carotid measurements in this study cohort, baseline CIMT values for the 116 subjects randomized to chloroquine or placebo were correlated with common variables associated with atherosclerosis. CIMT was positively correlated with age (r_s_ = 0.3963, P < 0.0001), systolic blood pressure (r_s_ = 0.3379, P = 0.0002), triglycerides (r_s_ = 0.2936, P = 0.0014), cholesterol (r_s_ = 0.3593, P < 0.0001), and LDL-C (r_s_ = 0.2590, P = 0.0050). There were no correlations with HDL-C, diastolic blood pressure, waist circumference, BMI, glucose, TNFα, IL-6, VEGF, or IL-17A (not shown).

**Table 3:** Baseline characteristics of subjects in the double-blind study

Variable	Chloroquine group	Placebo group	P value
Gender (F/M)	44/12	33/18	0.1108
Age	55 ± 12	55 ± 9	0.9999
Race (AA/NHW/H/NA)	9/45/1/1	6/45/0/0	0.4990
Smoking status			
Current	8	10	0.4341
Past	21	23	0.2962
Statin treatment	15	15	0.8310
Hypertension treatment	20	19	0.9999
Fish oil treatment	5	10	0.1632
Waist circumference	110.4 ± 10.5	108.9 ± 11.8	0.6538
BMI	36.2 ± 5.0	34.2 ± 5.0	0.0433*
SBP			
Screen	137.1 ± 14.3	140.2 ± 13.2	0.2385
Randomization	129.2 ± 11.4	134.0 ± 10.1	0.0237*
DBP			
Screen	82.8 ± 9.5	83.0 ± 9.7	0.9483
Randomization	78.3 ± 7.4	79.7 ± 7.2	0.3244
Glucose	5.48 ± 0.53	5.52 ± 0.64	0.6960
Triglycerides	1.84 ± 1.04	1.82 ± 0.93	0.9155
HDL-C	1.14 ± 0.26	1.14 ± 0.27	0.9603
Cholesterol	5.00 ± 0.85	5.18 ± 1.15	0.3834
LDL-C	3.04 ± 0.68	3.28 ± 0.82	0.1073

Data represent mean ± SD

There was no effect of chloroquine on CIMT after 12 months (Fig. 4b). Because chloroquine has a long half-life, imaging was repeated at 24 months, following a 12-month washout period. These results also showed no difference between groups. MRI studies were performed only at baseline and at 12 months. Chloroquine did not affect contrast enhancement, a pre-specified secondary endpoint of the study (Fig. 4c, P = 0.0697, paired two-tailed t test). Representative images of vessels at baseline and 12 months are shown in Fig. 4d. Carotid dimensions were also assessed by MRI. Chloroquine was associated with a decrease in common carotid artery luminal area (Fig. 4e, P = 0.0033) and diameter (Fig. 4f, P = 0.0019).

### Effects of chloroquine on blood pressure, lipids, and other variables

**Fig. 5:**
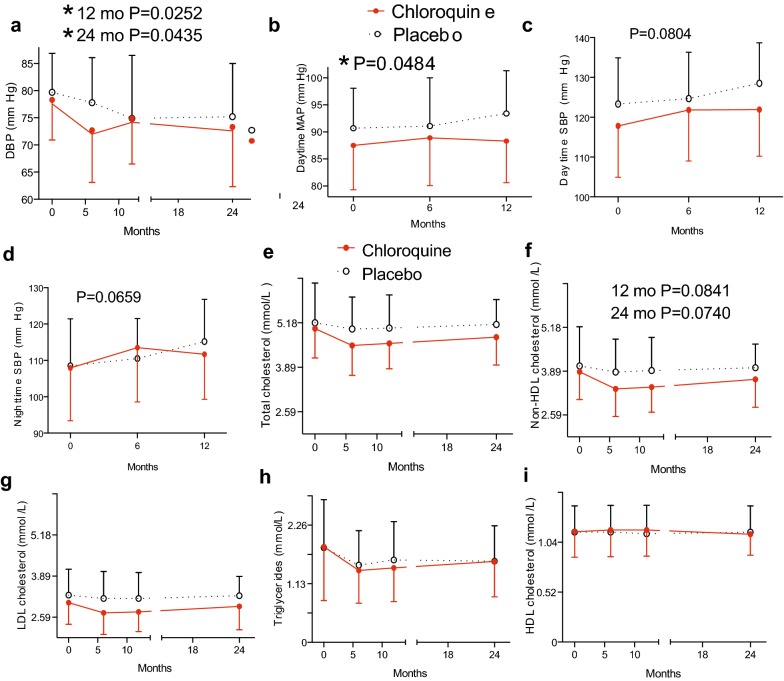
Blood pressure and lipid responses in the yearlong chloroquine trial. **a** Diastolic blood pressure over 24 months determined by conventional testing. **b**–**d** Blood pressures over 12 months, determined by an ambulatory monitoring device. **e**–**i** Lipids and lipoproteins over 24 months. P values were determined by mixed model treatment of repeated measures

Drug treatment over the first year was associated with a significantly lower diastolic blood pressure (P = 0.0252 at 12 months), results that were driven by a 4.9 mm Hg decrease at the 6 month time point (Fig. 5a). Ambulatory monitoring results (performed at baseline, 6 months, and 12 months but not at 24 months) were consistent with a beneficial effect of chloroquine on blood pressure. Drug treatment over the first year was associated with significantly lower daytime mean arterial pressure (Fig. 5b, P = 0.0484). Daytime (P = 0.0804) and nighttime (P = 0.0659) systolic pressures by ambulatory monitoring were not different (Fig. 5c, d). By mixed model testing for the interaction between group and time, there was no effect of chronic chloroquine on lipids. However, at 12 months, a pre-specified secondary endpoint, total cholesterol was 9% lower (Fig. 5e, P = 0.0089 by unpaired t test), non-HDL cholesterol was 12% lower (Fig. 5f, P = 0.0048 by unpaired t test), and LDL-C was 13% lower (Fig. 5g, P = 0.0038 by unpaired t test) in the chloroquine group. Glucose area under the curve (AUC) as well as HOMA-IR and the Matsuda index, measures of insulin sensitivity [24], were not different between groups (Table 4). There was no effect of chloroquine on insulin AUC, C peptide AUC, or the insulinogenic index (the difference between insulin at 30 min and 0 min divided by the difference between glucose at 30 min and 0 min) (Table 4), suggesting that chronic chloroquine treatment at this dose does not impact insulin levels in humans. Glucagon AUC, creatinine, ALT, and hematologic variables were not different between treatment groups (Table 4).

**Table 4:** Effects of chloroquine vs. placebo over 24 months (off either, i.e. washout, months 12–24)

Variable	Baseline	6 months	12 months	24 months	P value
Waist (cm)					
Chloroquine	110.4 ± 10.5	112.5 ± 12.6	112.6 ± 13.6	113.2 ± 16.6	0.5578 (12 m)
Placebo	108.9 ± 11.8	110.4 ± 13.8	109.8 ± 12.4	108.9 ± 15.9	
Weight (kg)					
Chloroquine	102.9 ± 16.7	102.9 ± 16.7	103.1 ± 19.2	105.9 ± 20.4	0.1323
Placebo	99.7 ± 18.5	98.7 ± 18.5	99.2 ± 17.8	100.5 ± 21.2	0.1909 (t test 24 m)
Fasting glucose					
Chloroquine	5.48 ± 0.53	5.36 ± 0.55	5.30 ± 0.51	5.58 ± 0.73	0.2741 (12 m)
Placebo	5.52 ± 0.64	5.52 ± 0.62	5.51 ± 0.71	5.63 ± 0.74	0.0930 (t test 12 m)
Glucose AUC					
Chloroquine	16.32 ± 2.46	15.94 ± 2.24	16.17 ± 2.58	16.97 ± 3.20	0.2537
Placebo	16.54 ± 3.49	16.87 ± 3.29	16.43 ± 3.34	17.50 ± 3.85	
Insulin AUC					
Chloroquine	1202 ± 681	1118 ± 542	1143 ± 603	1213 ± 716	0.2479
Placebo	1003 ± 524	1054 ± 588	995 ± 570	1022 ± 613	
C peptide AUC					
Chloroquine	7.46 ± 2.33	7.23 ± 2.10	7.16 ± 2.20	7.39 ± 2.43	0.2918
Placebo	6.49 ± 1.80	6.69 ± 2.06	6.46 ± 2.00	6.79 ± 2.06	
HOMA-IR					
Chloroquine	3.89 ± 2.23	3.85 ± 2.21	3.57 ± 2.12	3.72 ± 3.53	0.8421
Placebo	3.54 ± 2.03	3.64 ± 2.08	3.30 ± 2.23	3.29 ± 2.26	
Matsuda index					
Chloroquine	2.89 ± 1.72	2.88 ± 1.55	3.13 ± 2.31	3.43 ± 2.84	0.8997
Placebo	3.21 ± 1.89	3.07 ± 1.64	3.50 ± 2.14	3.56 ± 2.70	
Insulinogenic index					
Chloroquine	1.40 ± 1.75	1.17 ± 0.87	1.24 ± 0.87	1.12 ± 0.89	0.4988
Placebo	1.05 ± 0.77	1.03 ± 0.67	1.09 ± 0.88	1.13 ± 1.62	
Glucagon AUC					
Chloroquine	174.0 ± 63.5	152.3 ± 60.3	126.8 ± 43.3	ND	0.6126
Placebo	168.0 ± 60.6	138.7 ± 46.7	119.8 ± 36.2	ND	
Creatinine (μmol/L)					
Chloroquine	68 ± 14	67 ± 15	66 ± 15	ND	0.7859
Placebo	66 ± 16	65 ± 17	65 ± 17		
ALT					
Chloroquine	26 ± 16	26 ± 19	26 ± 15	ND	0.2056
Placebo	25 ± 12	27 ± 16	23 ± 9	ND	
WBC count					
Chloroquine	6.7 ± 1.8	6.3 ± 1.6	6.0 ± 1.5	ND	0.7249
Placebo	6.5 ± 1.7	6.3 ± 1.8	6.1 ± 1.9	ND	
Hemoglobin					
Chloroquine	13.6 ± 1.1	13.3 ± 1.1	13.2 ± 1.2	ND	0.8953
Placebo	13.8 ± 0.8	13.5 ± 0.9	13.5 ± 0.9	ND	

Data represent mean ± SD

P values determined by mixed model approach to repeated measures except where indicated

### Potential mechanistic mediators

**Fig. 6:**
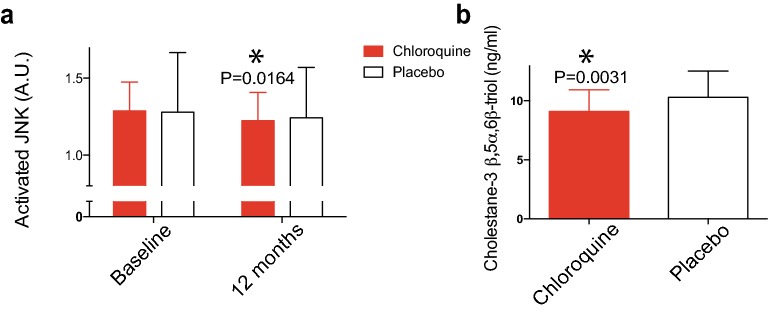
Effects of placebo and chloroquine on potential mechanistic mediators. **a** Activated JNK in circulating monocytes at baseline and 12 months. *P = 0.0164; P = 0.3343 for placebo; paired t tests. **b** Plasma concentrations of the oxysterol cholestane-3β, 5α, 6β-triol at 12 months. *P = 0.0031; unpaired t test

JNK is a stress kinase implicated in obesity-associated chronic inflammation [25]. JNK activation in monocytes, a pre-specified secondary endpoint, was significantly decreased in chloroquine-treated but not placebo-treated subjects (Fig. 6a). Reactive oxygen species activate JNK and have been implicated in the pathophysiology of obesity-associated conditions [26]. Plasma cholesterol oxidation products are elevated in diabetes and coronary artery disease [20]. Cholestane-3β, 5α, 6β-triol, one species that may reflect clinically relevant oxidative stress [20], was significantly decreased in the chloroquine group at 12 months (Fig. 6b).

### Adverse events

For the dose escalation study, there were no serious adverse events (Table 5). Not unexpectedly given the rigors of a series of clamps, there were several study-related events. Of adverse events that were not directly study-related, infections were the most common.

**Table 5:** Adverse events in the dose escalation study

Adverse event	Subjects (%)
Total subjects with any event	24 (69)
Subjects with a serious adverse event	0 (0)
Total adverse events	57 (100)
Study related	14 (25)
IV placement difficulties	1 (1.8)
IV infiltration	1 (1.8)
Phlebitis	1 (1.8)
Skin reaction to tape	1 (1.8)
Extremity erythema due to BP cuff	1 (1.8)
Vasovagal episode with IV placement	1 (1.8)
Incorrect stable isotope administered	1 (1.8)
Nausea	3 (5)
Anemia	4 (7)
Not study related	43 (75)
Infectious	15 (26)
Allergy/immunology	3 (5)
Musculoskeletal	8 (14)
CNS	3 (5)
Psychiatric	5 (9)
Cardiovascular	2 (4)
GI	4 (7)
Metabolic	3 (5)

Parentheses denote %

For the double blind study, there were 7 serious events. In the placebo group, one subject presented with a new diagnosis of prostate cancer. One subject underwent a right knee replacement. In the chloroquine group, one patient developed transverse myelitis presenting with left leg weakness that subsequently improved but did not completely resolve. One subject underwent carotid endarterectomy for asymptomatic near-total carotid occlusion detected at 6 months. One subject was hospitalized for a cutaneous staphylococcus infection that resolved with appropriate therapy. One subject was treated for acute appendicitis, stopped the study medication for 1 week then resumed without incident. One subject was found to have a positive PPD without evidence of pulmonary tuberculosis.

Total adverse events were similar between groups (Table 6). There were more infectious events in those randomized to chloroquine but this was not significant (51 vs. 42, P = 0.0609). No subject in either study developed retinal pigmentary changes that may occur with chloroquine. For the double blind study, observers masked to the treatment assignment examined each subject at 0, 6, 12, and 24 months. Ocular adverse events were not significantly different between treatment groups (Table 6).

**Table 6:** Adverse events in the double-blind study

Adverse event	Chloroquine subjects (%)	Placebo subjects (%)	P value
Total subjects with any event	44 (76)	39 (67)	0.4107
Subjects with a serious adverse event	5 (9)	2 (3)	0.4385
Total adverse events	109 (100)	105 (100)	–
Infectious	51 (47)	42 (40)	0.0609
Allergy/immunology	5 (5)	10 (10)	0.2681
Musculoskeletal	17 (16)	15 (15)	0.8357
CNS	5 (5)	3 (3)	–
Psychiatric	5 (5)	6 (6)	–
Hematologic/neoplasm	2 (2)	2 (2)	–
Cardiovascular	2 (2)	3 (3)	–
GI	3 (3)	3 (3)	–
GU	2 (2)	2 (2)	–
Metabolic	1 (1)	6 (6)	–
Miscellaneous	0 (0)	3 (3)	–
Ocular	16 (15)	10 (10)	0.2654
Ocular details			
Retinal	3	3	–
Lens	4	2	–
Vision	2	0	–
Infectious	2	0	–
Glaucoma	2	2	–

Parentheses denote %

P values determined using contingency tables

## Discussion

We tested the hypothesis that the anti-malarial drug chloroquine, which activates the kinase ATM, improves insulin sensitivity and decreases atherosclerosis in humans with metabolic syndrome. In mice, low dose chloroquine improves insulin sensitivity, decreases atherosclerosis, and suppresses activation of JNK in macrophages through pathways that require the presence of ATM [6]. The absence of ATM in mice affects hepatic insulin sensitivity and promotes atherosclerosis, suggesting the involvement of processes that link hepatic insulin resistance and vascular disease. Accordingly, we translated these findings to humans by conducting two trials, the first to determine if chloroquine improves insulin sensitivity in the liver, and the second to determine if chloroquine decreases CIMT.

The overall results were disappointing. Hyperinsulinemic euglycemic clamp studies demonstrated a dose response relationship between chloroquine administration and hepatic insulin sensitization but the effect was modest. Treatment with chloroquine for 1 year (using the lowest dose found to increase hepatic insulin sensitivity) had no effect on the primary endpoint of CIMT and no beneficial effects on MRI imaging. One limitation of the study is that it may have been underpowered to detect a difference in CIMT. Sample size estimates at the time of initiation of these studies were based on reports showing that insulin sensitizers had effects on CIMT with groups of 31–57 subjects [27–30], but subsequent work indicated the need for larger sample sizes [31]. Taken together, our findings suggest that chloroquine will not be clinically useful for the treatment of metabolic syndrome.

There were positive results from our studies. Chronic administration of chloroquine lowered blood pressure, decreased activation of JNK in circulating monocytes, and decreased concentration of a cholesterol oxidation product that reflects oxidative stress. These findings suggest that chloroquine decreases systemic inflammation and improves some components of the metabolic syndrome. The inhibition of JNK is known to enhance insulin sensitivity and decrease atherosclerosis in animals [32, 33]. Oxidative stress activates JNK, and recent studies implicate ATM, which is activated by chloroquine, in modulation of reactive oxygen species generation [34]. Metformin, the most widely used insulin sensitizer, appears to work through redox-dependent mechanisms in liver [35]. Variants in the ATM gene alter the glycemic response to metformin in humans [36]. These findings raise the possibility that chloroquine induction of ATM activity modifies redox signaling to decrease insulin resistance, but in humans these effects are small.

Hydroxychloroquine, closely related to chloroquine, improves glycemic control in poorly controlled type 2 diabetes in humans [37] and may improve glucose metabolism in human prediabetes [38]. Its use in rheumatoid arthritis is associated with a reduced risk for developing diabetes in large observational studies [39, 40], and its administration to healthy obese subjects without the metabolic syndrome increases the Matsuda index of insulin sensitivity [41].

Chloroquine was well tolerated. Subjects in the dose escalation study received 7.2 g and those in the yearlong study 29.2 g. Retinopathy is a serious but rare adverse effect of chloroquine; our subjects had regular eye exams and retinopathy was not detected.

## Conclusions

Low dose chloroquine modestly enhanced hepatic insulin sensitivity but did not affect CIMT. Despite some positive effects on secondary endpoints including the demonstration of suppression of the stress kinase JNK, overall results suggest that chloroquine will not be useful for the treatment of metabolic syndrome in humans.

## Data Availability

The datasets used and analyzed in the current study are available from the corresponding authors on reasonable request. All data generated during this study are included in this published article.

## Abbreviations

ALT: alanine aminotransferase
ANOVA: analysis of variance
AUC: area under the curve
ATM: ataxia telangiectasia mutated
BMI: body mass index
CIMT: carotid intima-media thickness
CLIA: Clinical Laboratory Improvement Amendments
COPD: chronic obstructive pulmonary disease
CRP: C-reactive protein
DBP: diastolic blood pressure
DNA: deoxyribonucleic acid
G6PD: glucose-6-phosphate dehydrogenase
HDL: high density lipoprotein
HIV: human immunodeficiency virus
HOMA-IR: homeostasis model assessment-insulin resistance
IL-6: interleukin 6
IL-17A: interleukin 17A
IV: intravenous
JNK: c-Jun-N-terminal kinase
LDL: low density lipoprotein
LDL-C: low density lipoprotein cholesterol
MAP: mean arterial pressure
MRI: magnetic resonance imaging
NEFA: non-esterified fatty acid
NHLBI: National Heart, Lung, and Blood Institute
OGTT: oral glucose tolerance test
SBP: systolic blood pressure
SD: standard deviation
SE: standard error
TNFα: tumor necrosis factor alpha
VEGF: vascular endothelial growth factor
